# Climate variability impacts on rice production in the Philippines

**DOI:** 10.1371/journal.pone.0201426

**Published:** 2018-08-09

**Authors:** Malte F. Stuecker, Michelle Tigchelaar, Michael B. Kantar

**Affiliations:** 1 Center for Climate Physics, Institute for Basic Science (IBS), Busan, Republic of Korea; 2 Pusan National University, Busan, Republic of Korea; 3 Department of Atmospheric Sciences, University of Washington, Seattle, WA, United States of America; 4 Department of Tropical Plant & Soil Sciences, University of Hawaii at Manoa, Honolulu, HI, United States of America; Centro de Investigacion Cientifica y de Educacion Superior de Ensenada Division de Fisica Aplicada, MEXICO

## Abstract

Changes in crop yield and production over time are driven by a combination of genetics, agronomics, and climate. Disentangling the role of these various influences helps us understand the capacity of agriculture to adapt to change. Here we explore the impact of climate variability on rice yield and production in the Philippines from 1987–2016 in both irrigated and rainfed production systems at various scales. Over this period, rice production is affected by variations in soil moisture, which are largely driven by the El Niño–Southern Oscillation (ENSO). We found that the climate impacts on rice production are strongly seasonally modulated and differ considerably by region. As expected, rainfed upland rice production systems are more sensitive to soil moisture variability than irrigated paddy rice. About 10% of the variance in rice production anomalies on the national level co-varies with soil moisture changes, which in turn are strongly negatively correlated with an index capturing ENSO variability. Our results show that while temperature variability is of limited importance in the Philippines today, future climate projections suggest that by the end of the century, temperatures might regularly exceed known limits to rice production if warming continues unabated. Therefore, skillful seasonal prediction will likely become increasingly crucial to provide the necessary information to guide agriculture management to mitigate the compounding impacts of soil moisture variability and temperature stress. Detailed case studies like this complement global yield studies and provide important local perspectives that can help in food policy decisions.

## Introduction

Rice–which provides nearly half the calories for half the world’s population [1;2]–is a key crop for the Philippines: it is a staple food (with >110 kg/person/year consumption, [3], http://irri.org/rice-today/nourishing-a-nation), the sixth highest per capita consumption in the world), as well as a major source of income (rice production valued at ~6 billion U.S. dollars in 2015; [4]). The Philippines produces approximately 3% of the world’s rice in both “lowland” flooded transplanted paddies and “upland” rainfed direct seeded areas [5]. As such, understanding what drives changes in rice production in the Philippines is essential for meeting current and future food security [6;7]. Variations in crop yields can be explained by either endogenous drivers, such as genetics (including breeding methods–pure line, synthetic, hybrid) and agronomy (including technology–use of fertilizer, irrigation, machinery) [6;8;9], and exogenous forcing such as climate variability, which has been reported to decrease the influence of genetics [10]. The role of climate is becoming increasingly important due to anthropogenic climate change, which could drastically change local environments, damage yields [11;12], and influence the yield stability of staple crops [13;14]. Here we assess how current and future climate variability influences the various modes of rice production in the Philippines.

Continuing to feed a growing world population expected to reach ~9 billion by 2050 [15] while faced with a changing climate is a tremendous challenge. To date, global food production has steadily increased through innovations in agricultural technology (improved practices and genetics). The Philippines has mirrored global trends, with population increasing from ~26 million in 1960 to ~101 million in 2015, and rice production increasing from ~3.9 million tonnes in 1961 to ~19.0 million tonnes in 2014. This large improvement has been due to increased yields (production per unit area) and increased acreage being placed into production [16]. However, it is unclear whether it will be possible to sustain increasing production into the future [17], and if the changing land use patterns for agriculture are sustainable [18].

The Philippines is a large and spatially heterogeneous country, consisting of 7107 islands divided into 18 political regions and 81 provinces. There are four major climate regimes: 1) distinct wet monsoon and dry season, 2) no distinct dry season but a strong wet monsoon season, 3) intermediate between type 1 and 2, where there is a short wet monsoon and short dry season, and 4) an even distribution of rainfall throughout the year [19]. Planting dates vary between regions based largely on differences in climate (S1 Table). While rice in the Philippines is grown throughout the year (S1 Table), the largest production share is grown during the wet season. Due to this diversity of planting and harvesting, the government of the Philippines takes annual, semester, and quarterly statistics on rice production and harvested areas. Farms in the Philippines are generally small (less than two hectares on average; [20]), which may limit the implementation of advanced farming technologies. Currently, irrigated paddy rice accounts for 60% of total production [21], with the remainder grown as upland directly seeded rice.

In the Philippines, the dominant climate influence on inter-annual timescales is from the El Niño–Southern Oscillation (ENSO). ENSO has pronounced effects on global rainfall and temperature variability, particularly in the Indo-Pacific region [22;23;24]. It has been shown that this inter-annual climate variability can drastically impact crop yields and production globally [14;25;26]. In the tropical western Pacific region, El Niño events (the warm phase of ENSO) generally have a negative effect on farming. Specifically, El Niño induced droughts in the western Pacific have detrimentally affected Indonesian rice production [27], with worsening effects projected in response to greenhouse gas forcing [28]. Previous work on ENSO in the Philippines has shown that dry-season rice production is negatively impacted by El Niño on Luzon Island [25]. Additionally, tropical cyclones are a source of weather variability that is strongly seasonally modulated and exhibits localized impacts, suggesting that climate-yield and climate-production relationships need to be evaluated regionally and on sub-annual timescales.

An important limiting factor to increased food production in response to population growth and dietary shifts in the next century is the ability of crops to respond to climate variability, for instance soil moisture, surface temperatures, and the frequency of severe storms [29;30]. Studies of climate impacts on crops typically either use process-based crop models, or evaluate the statistical relationship between crop production and climate variability in the past. Here we use this latter method to evaluate the impact of climate variability on rice production in the Philippines in different spatial and temporal contexts, and compare the range of past climate variability to projected future climate change to assess whether these relationships can be expected to hold in the future. We find that using a finer temporal and spatial resolution provides a more detailed understanding of climatic drivers of rice production, especially for upland (rainfed) rice, which is significantly impacted by ENSO through modifications in soil moisture. By the end of the century, temperatures will likely exceed present-day ranges, and will thus become an additional limiting factor to rice yield and production.

## Materials and methods

### Data acquisition

Rice production data from 1987–2016 were obtained from the Philippine government statistic authority (http://countrystat.psa.gov.ph/) for each political region and nationwide. Area harvested (hectares) and production (metric tonnes) data were collected from each political region and for the whole country for each quarter and year, for both irrigated and rainfed rice production. Missing data (where survey data was not complete) were linearly interpolated for each region (harvested area and production for rainfed systems) on the quarterly data (less than 1% of the data were missing). No values were missing for irrigated systems. Yield (tonnes per hectare) was calculated by dividing production by area for each quarter from 1987–2016.

To explore the ecological tolerance of rice we obtained the locality information of accessions stored in gene banks worldwide from https://www.genesys-pgr.org for tropical localities (from 23.5°S-23.5°N). From geo-referenced coordinates, we obtained surface temperature data for tropical rice from the WorldClim database at 30 arc seconds resolution [31], which were used to explore the climatic space inhabited by tropical rice.

### Yield normalization

We created continuous time series of production and yield (rainfed and irrigated) for each aggregated political region. To remove the effect of yield increases due to breeding methods, we removed a ~7 year (27 quarters) running mean from each continuous time series and afterwards removed the residual total mean to construct an anomalous time series with zero mean. The results were qualitatively stable to the choice of the running mean window size (a 5 year window was also tested, data not shown). These normalization timescales are commonly used in the literature [11;32] and correspond to a normal life cycle of a rice genotype used in farming [33].

### Climate data

To calculate climate anomalies, we removed both the annual cycle (1987–2016 climatology) and the linear trend from each of the climate variables used. ENSO variability was characterized using the Niño3.4 (N3.4) index, which is calculated as the area averaged sea surface temperature anomalies from HadISST1 [34] in the region 170°W-120°W and 5°S-5°N. Soil moisture data were obtained from CPC (version 2) at 0.5° horizontal resolution [35]. Surface air temperature (2m) was obtained from the ERA-Interim reanalysis [36] on a 0.125° horizontal grid. For the global warming projections (see below), the present-day reference temperatures were obtained from the CRU TS version 3.23 dataset, which presents monthly data from the period 1901–2014 on a 0.5° horizontal grid [37]. To evaluate crop-climate relationships at the different spatial scales, climate data were either spatially averaged for the entire Philippines (here defined by the geographical region 117°E-128°E, 4°N-22°N) or the respective regions (see S1 Table).

### Climate projections

Future climate projection data were obtained from the CMIP5 database [38] for the business-as-usual scenario RCP 8.5. Monthly output was obtained from eighteen climate models and interpolated using bilinear interpolation to a 0.5° resolution common grid. For the 2°C and 4°C warming targets, we first constructed the canonical global warming temperature pattern [39] for each of the eighteen models by taking the difference in monthly climatology between the 2080–2099 and 1980–1999 time periods, normalized by the global, annual mean temperature change. The future climate projections are then calculated by adding the change in each (2°C or 4°C warmer) model climatology to the observed (1911–2010) climate history, thus preserving the present-day interannual temperature variability [40].

### Correlation analysis

We utilize standard correlation analysis to investigate the relationships between the respective climate variables and rice production and yield. For these relationships, we consider seasonal anomalies to be independent from anomalies in the same season of the previous and following years, which leads to an effective sample size of 30 (number of years). For all spatial maps that show temporal correlation coefficients in shading for the different geographical regions, an absolute value of the correlation coefficient of ~0.31 is statistically significant at the 90% confidence level using a two-tailed t-test (df = 28). Thus, we are not showing any correlations below an absolute value of 0.3 (white shading) in these maps.

## Results

### National-level data

**Fig 1:**
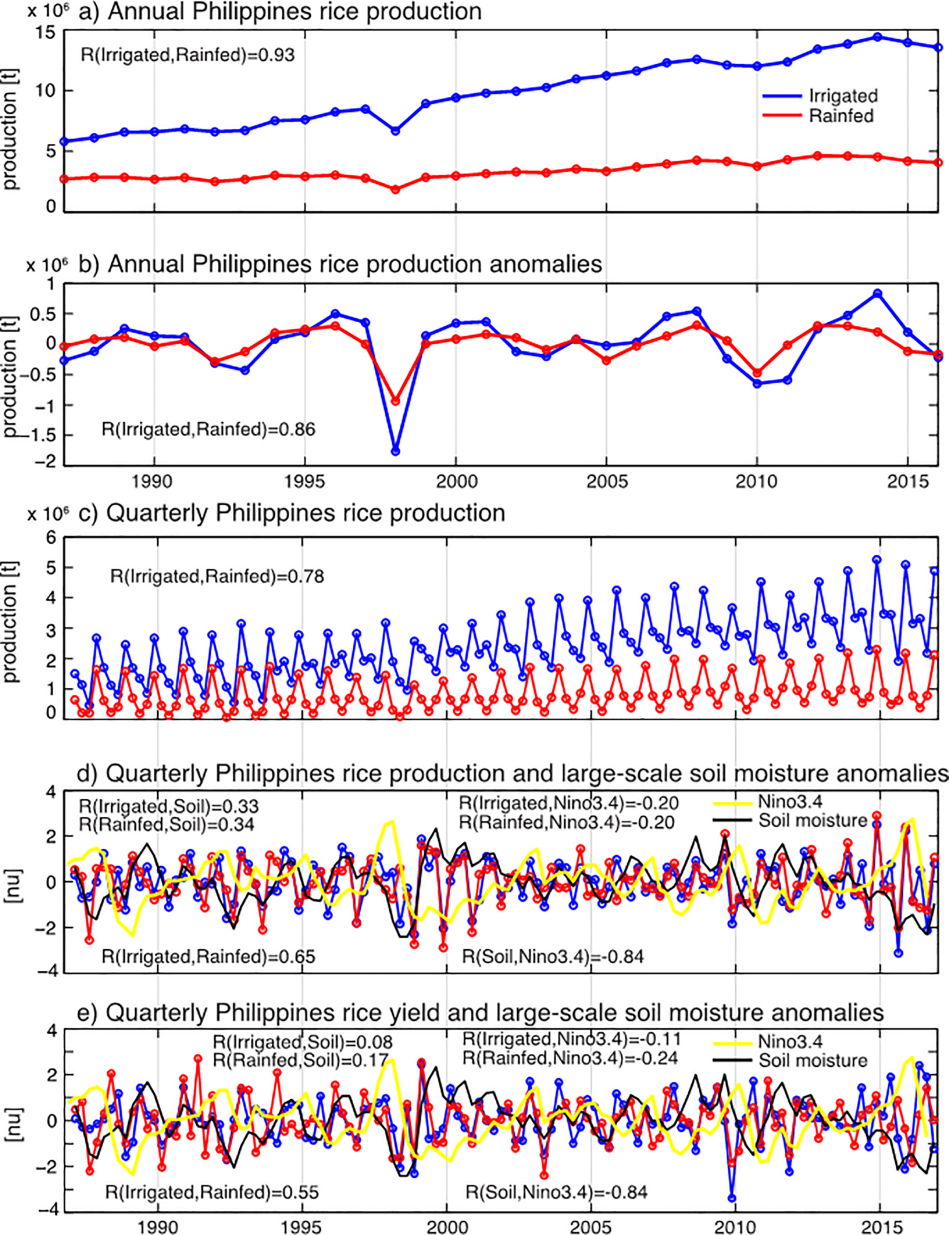
National-level rice production in the Philippines from 1987–2016: Irrigated (blue) and rainfed (red) farming techniques. A) Annual rice production in the Philippines; B) Annual rice production anomalies (with regard to a 7 yr moving average); C) Quarterly rice production; D) Normalized quarterly rice production anomalies (the annual cycle is removed and the anomalies are with regard to a 7 yr moving average); E) Normalized quarterly rice yield anomalies. Additionally, d) and e) show the quarterly normalized soil moisture anomalies averaged from 117°E-128°E and 4°N-22°N (black line) and the normalized Niño3.4 index (yellow line). In all panels, R indicates instantaneous correlation except for the correlation coefficients in D) and E) between rice production/yield and soil moisture, which are given for a 3 months lead time of soil moisture, between Niño3.4 and soil moisture for a 4 months Niño3.4 lead time, and between Niño3.4 and rice production/yield for a 7 months Niño3.4 lead time.

Irrigated rice production in the Philippines has almost tripled over the past thirty years, while rainfed rice production has seen a much smaller growth (Fig 1A). Over this period, yields for both production systems have increased steadily (S1A Fig). Besides this long-term trend, annual rice yields at the national level have been fairly stable over this period, with irrigated paddy rice production having only six yield anomalies exceeding one standard deviation (absolute anomaly of 0.09 [t ha^-1^], which corresponds to ~2.5% of the annual long-term mean in irrigated), while rainfed upland rice crops exhibited eight yield anomalies exceeding one standard deviation (absolute value of 0.07 [t ha^-1^], which corresponds to ~2.9% of the annual long-term mean in rainfed; S1B Fig). Relative anomalies in total rice production (Fig 1B) are larger than those in yield, implying that the effects of climate variability are compounded through both yield and harvested area changes. As a result of the frequent occurrence of natural disasters in the Philippines, production losses are often manageable and built into farm management [41]. Notable exceptions are 1998 –with two typhoons–and 2010 –with four typhoons, an earthquake and a flood–which both saw large negative production anomalies [42].

Aggregating the yield and production data on an annual time scale potentially masks seasonal modulations of both the large-scale climate variability [24] and crop-climate relationships [25]. As a result, quarterly production and yield anomalies [Fig 1D and 1E] show more variability than the annual data. Rainfed and irrigated rice production anomalies are substantially less correlated with production in the quarterly data (R = 0.65, significant at the 99% confidence level with df = 28) than in the annual time series (R = 0.86, significant at the 99% confidence level with df = 28). About 10% of variance in anomalous rice production on the national level is related to soil moisture variability, which is strongly negatively correlated with the Niño3.4 index (Fig 1D). This reduction of rice production during El Niño events is qualitatively similar to the results of global analyses [26]. While the correlation coefficients between soil moisture anomalies and rice production anomalies are approximately the same for irrigated (R = 0.33, significant at the 90% confidence level with df = 28) and rainfed (R = 0.34, significant at the 90% confidence level with df = 28) rice production, when looking at yield anomalies the correlation is higher for rainfed than for irrigated systems (Fig 1E). This shows that, as expected, irrigation can counter much of the plant physiological response to soil moisture changes (as measured by rice yield), but decisions on planting area (as included in rice production) remain sensitive to water availability [25].

### ENSO impacts on soil moisture

**Fig 2:**
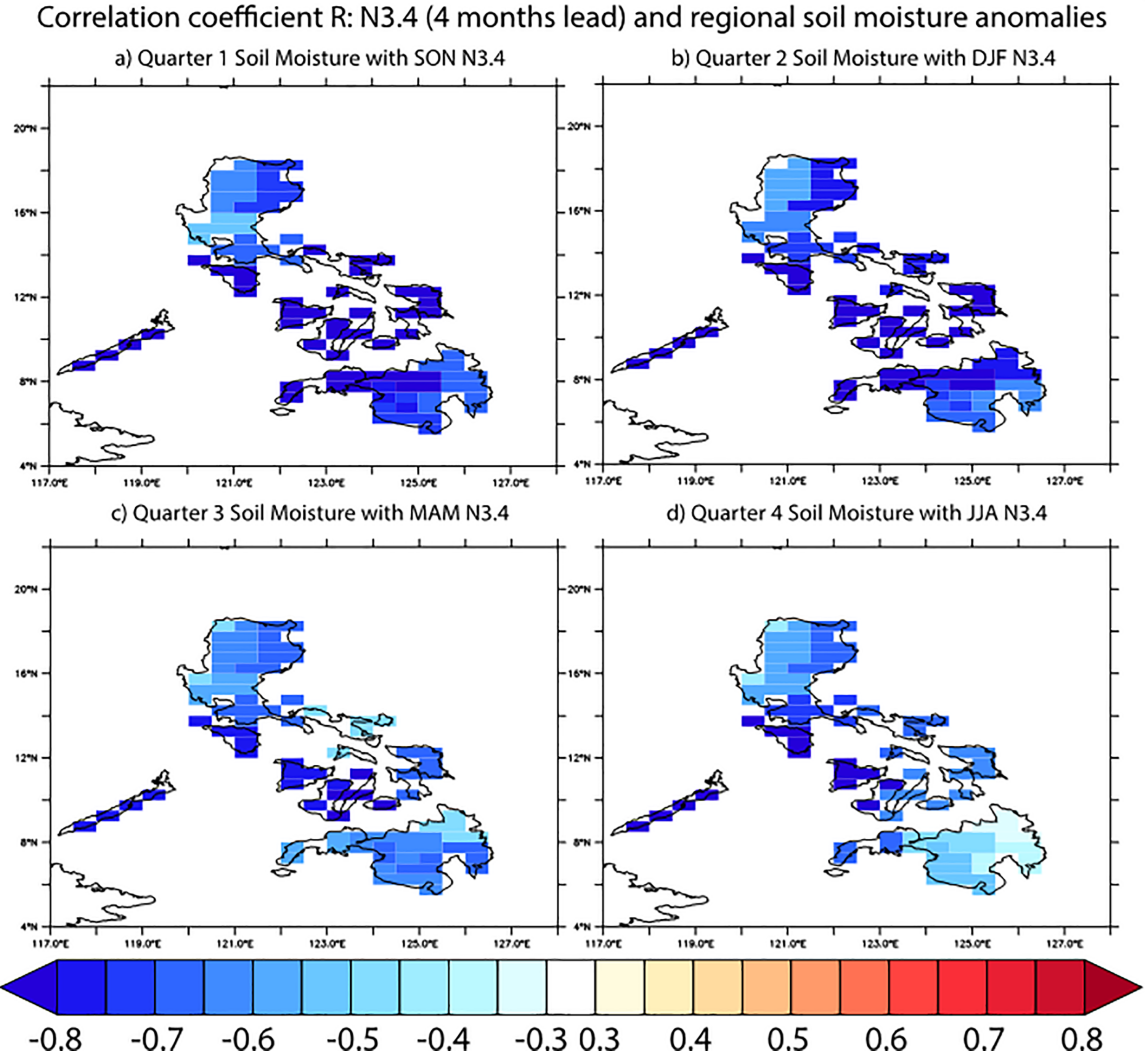
Correlation coefficient R between soil moisture anomalies and Niño3.4 index, using a 4 months lag (e.g., December 1986-February 1987 N3.4 with April 1987-June 1987 soil moisture).

On a regional scale as well as on the national level, the correlation between the Niño3.4 index and soil moisture anomalies in the Philippines is negative (Fig 2), i.e., El Niño events lead to dry conditions in all parts of the country. Interestingly, the correlation between ENSO and soil moisture decreases in the third and fourth quarters (Fig 2). One factor might be that in the summer season rainfall variability is dominated by tropical cyclone activity [43]. While tropical cyclone activity can be modulated by large-scale climate variability such as ENSO, it can be considered a mostly stochastic process on climate timescales. This wet season (Quarters 3 and 4) is also the season when most rice is planted (Fig 1C), indicating that wet-season rice production may be largely decoupled from ENSO variability [25].

### Regional crop-climate relationships

**Fig 3:**
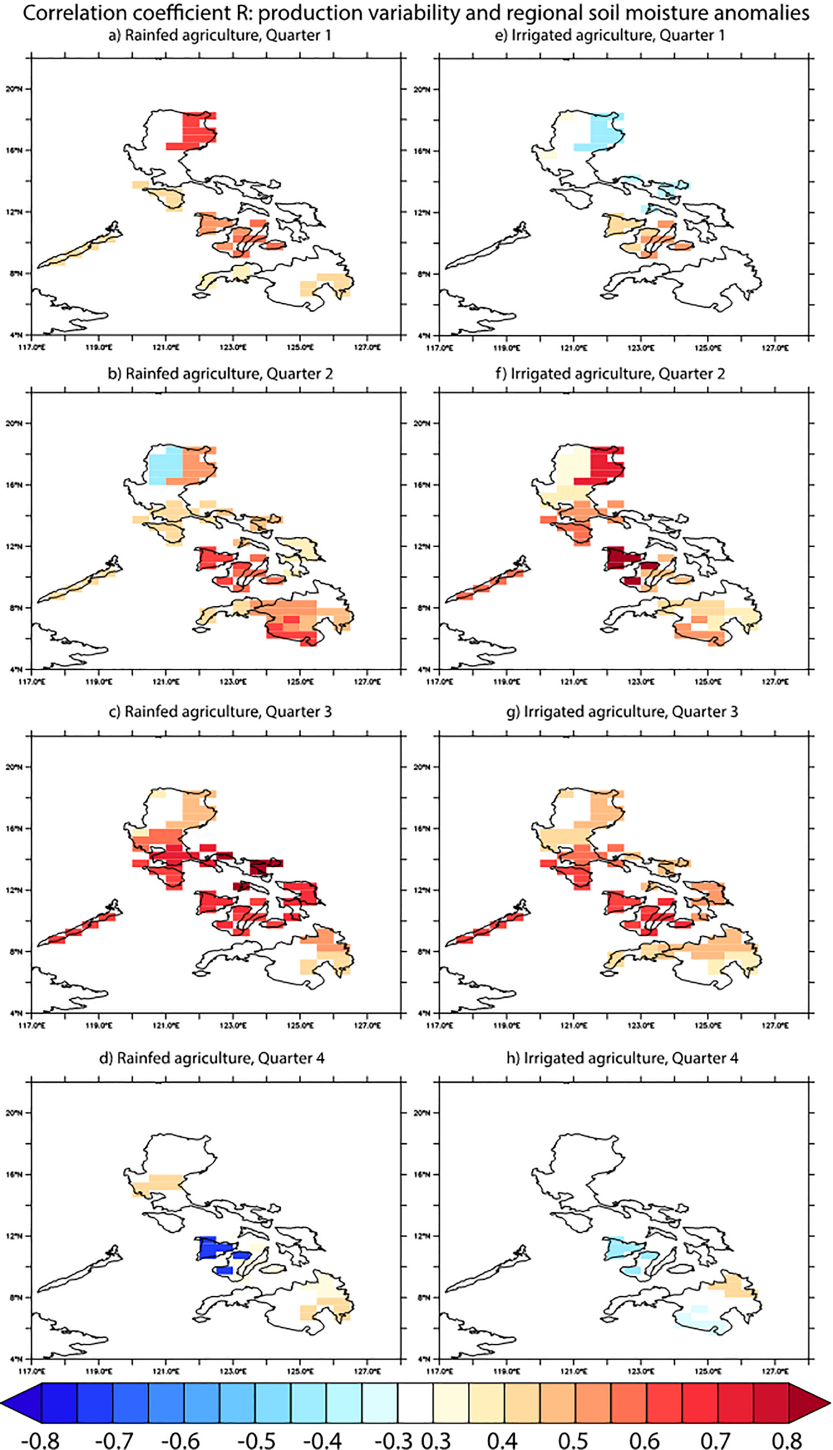
Correlation coefficient R between quarterly rice production and soil moisture anomalies in the previous quarter. The annual cycle is removed and production anomalies are with regard to a 7 yr moving average. The soil moisture data are area averaged for each political region corresponding to the rice production data.

Rice in the Philippines is in the field for 90–110 days, so that planting decisions are made about three months before harvest [25]. Looking at the lagged correlation between rice production and soil moisture (soil moisture leading by one quarter, Fig 3), in most seasons, soil moisture anomalies in the previous quarter are significantly correlated with production variability, with higher soil moisture usually associated with increased rice production. Locally, seasonal correlations can be much higher than the national-level data (Fig 1D). A notable exception to this is Quarter 4, when correlations between these two variables are small, or even negative (Fig 3). Production in this quarter is the highest of the year (Fig 1C) and represents the wet-season crop. Mean soil moisture conditions during the preceding quarters are high, so that variability in soil moisture does not affect rice planting or yield that much, while the typhoons that often impact the summer season (Q2-Q3) can lead to detrimental flooding in these quarters [43]. This is in accordance with an analysis of Luzon Island in the Northern Philippines (eight of eighteen regions; [25]), an area where both mean production (S2 Fig) and mean yields (S3 Fig) are high.

**Fig 4:**
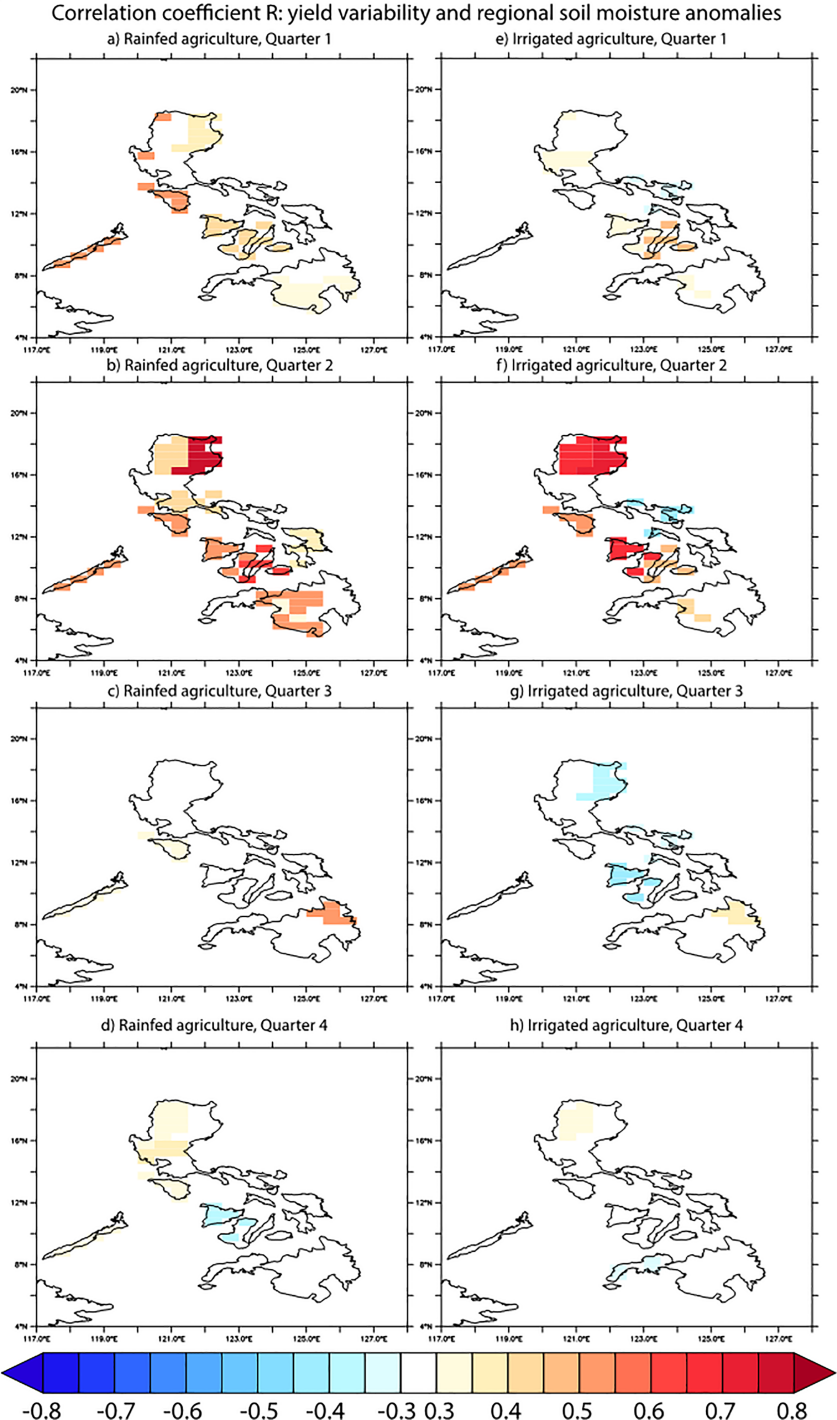
Correlation coefficient R between quarterly rice yield and soil moisture anomalies in the previous quarter. The annual cycle is removed and yield anomalies are with regard to a 7 yr moving average. The soil moisture data are area averaged for each political region corresponding to the rice yield data.

Total rice production in any given region is a function of the crop area harvested, the crop yield per unit area, and the number of crops harvested per year. Climate variability influences all of these variables. In Quarter 3, when correlations between soil moisture and total rice production are strongly positive in most regions (Fig 3), there were few locations with significant correlations between previous-quarter soil moisture and rice yield (Fig 4). This means that in this season, soil moisture anomalies might mostly drive planting decisions (i.e., which areas are brought into production), without strongly affecting plant development. During the dry season (Quarters 1 and 2) on the other hand, there are also significant regional correlations between soil moisture and rice yields, implying that climate variability in this season affects both plants and planting decisions. Mean climatological soil moisture conditions thus strongly affect the rice production response to climatic forcing. In contrast to soil moisture, in most regions temperature variability has a much lower correlation with rice yields. In some regions however, ENSO-induced temperature and precipitation changes have an effect in the same direction: El Niño events usually result in dry and hot conditions in the Philippines, which both are associated with a decrease in yield (S4 Fig). As we have seen, ENSO is driving a significant part of soil moisture variability in the Philippines which is correlated with rice production variability. Therefore, the predictive skill for ENSO that is seen in operational seasonal forecast models [44] up to several seasons ahead translates into important information for agriculture management in the Philippines and the possibility to mitigate some of the ENSO-induced effects on rice yields.

The correlation between rice production anomalies in upland rainfed and lowland irrigated systems is stronger in annual data (Fig 1B) than in the quarterly data (Fig 1D). On a regional level, there is a differential response to climate forcing between these two different management systems. For rice yield in particular, the response to soil moisture changes is, not unexpectedly, stronger for rainfed than for irrigated crops (Fig 4). Previous work found that on a global scale as well, yield losses during El Niño events are greater in rainfed areas compared to irrigated regions [26]. This shows that irrigation can provide a potentially useful management tool to mitigate climate impacts on rice production in the Philippines. At the same time soil moisture conditions are a direct proxy for local water availability–a major limiting factor for crop yield and production [45]–which could explain the correlations seen between irrigated rice yields and soil moisture anomalies in Quarter 2 (Fig 4F).

When looking at specific regions of high rice production (S2 Fig) on Luzon Island (large island in the northern Philippines that includes the regions Cagayan and Central Luzon) and Mimaropa (Southwestern islands within the Philippines), production and yield responses to soil moisture anomalies are not always consistent between these areas (Figs 3 & 4). Mimaropa exhibits one of the most consistently positive correlations between soil moisture anomalies and crop output in the Philippines, both in terms of total production and crop yield, and in rainfed and irrigated systems alike. In Central Luzon on the other hand, the response is more variable, and correlations are generally low for rice yields. Negative correlations between soil moisture and yield or production in some quarters and regions may reflect the damaging impact of flooding on rice, which happens fairly frequently [42]. Due to this nonlinear impact of rainfall on rice yield (i.e., an increase of rainfall can lead to either positive or negative rice yield depending on thresholds in the system), the actual yield variance explained by climate might be larger than suggested by linear correlation analysis, which should be explored further in future studies.

### Sensitivity to climate in the future

As we have shown here, climate-induced rice production variability in the Philippines over the past three decades has mostly been related to soil moisture changes, which in turn were associated with large-scale inter-annual rainfall variability caused by the El Niño–Southern Oscillation. This is in line with previous studies that show that although rice is grown over a large environmental range in both temperate and tropical areas [46], more variance in yield in tropical areas is usually due to precipitation (and thus also soil moisture) rather than temperature. Generally, tropical environments have relatively small variability in temperature, so other factors such as solar radiation, precipitation, or soil nutrient availability have a larger impact on crop production [47]. However, this particular expression of crop sensitivity to large-scale climate may fundamentally change in a warming climate [48].

**Fig 5:**
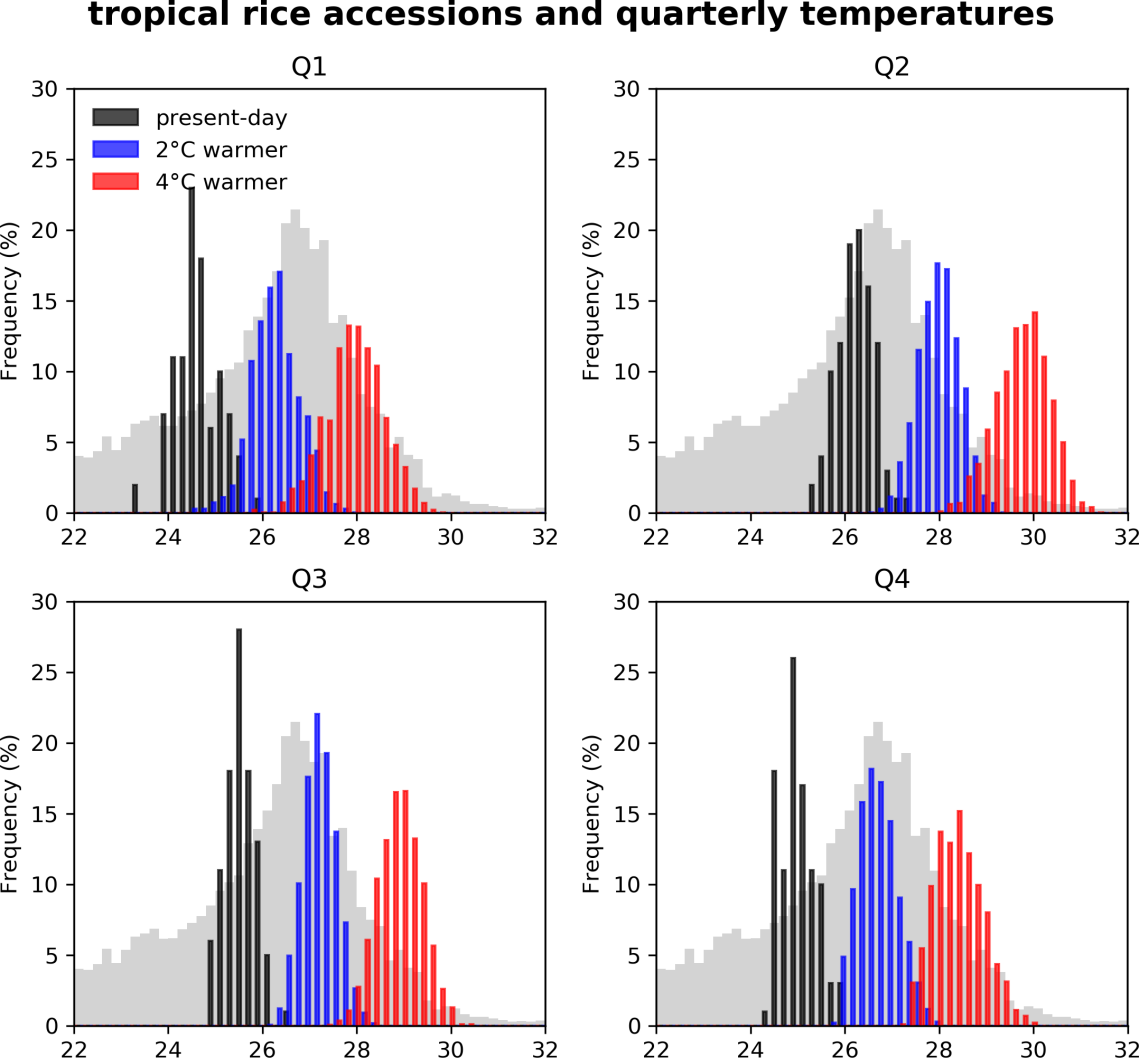
Histograms of quarterly temperatures averaged over the Philippines (black) observed from 1911–2010, (blue) projected with 2°C global warming, and (red) projected with 4°C global warming. The variance in future temperatures represents inter-model spread and present-day interannual variability. Occurrence points of rice in the tropics (23.5°S–23.5°N) using quarterly data are plotted in grey, with frequencies rescaled by a factor of 4. Rice location data were downloaded from Genesys PGR [78].

In the Philippines, temperatures year-round are currently within the range of favorable growing conditions for rice (Fig 5). Despite the fact that we see a significant proportion of variance explained by ENSO-mediated soil moisture variability, in the future the effect of temperature is likely to become increasingly important: If greenhouse gas emissions continue unabated, by the end of the century summers in the Philippines will be warmer than during the historical record [12]. Fig 5 shows the year-to-year variability in present-day quarterly temperatures, and how this is projected to change with 2 and 4°C of global warming. Over the past century, quarterly temperatures averaged over the Philippines never exceeded 27°C. With 2°C of global warming, median quarterly temperatures would be outside of the present-day range. With 4°C of global warming, year-to-year temperature variability will be entirely above the range of present-day variability. The effects of this will be particularly impactful during the dry season in Quarter 2, when temperatures are already high and there is low capacity for mitigation through soil moisture.

Under business-as-usual emissions (RCP8.5), the global mean temperature is projected to increase by 2°C as early as 2042, with a median prediction of 2055, and by 4°C between 2075 and 2132. Even in an emissions scenario aiming to stabilize greenhouse gas concentrations by mid-21st century (RCP 4.5), global mean temperature could rise by 2°C as early as 2052 [40]. Based on the temperature projections for these global warming targets, the Philippines is thus likely to see a fundamental shift in the climate–rice relationship over the course of the next few decades. This analysis focuses only on seasonal-mean temperature projections. However, the average precipitation, inter-annual climate variability, and the frequency of extremes may change as well, but projections for these are much more uncertain.

## Discussion

Regional and quarterly data of climate variability and rice production in the Philippines show that ENSO-induced changes in soil moisture are a major source of climate-driven production variability, especially during the dry season. Wet-season soil moisture changes seem to be more stochastically driven, and therefore more independent from large-scale climate forcing such as ENSO. During this main growing season background soil moisture conditions are high, so factors other than climate drive planting decisions and crop yields. The sensitivity to climate variability is higher in upland rainfed systems than in lowland irrigated systems, and varies strongly by region.

Regional differences in crop-climate relationships could be partly explained by differences in soil type, which determine water-holding capacity and thus soil moisture content and cropping patterns. Other factors that contribute to regional differences include different rice variety choices, different management practices (fertilization, mechanization, planting date, post-harvest storage), as well as different market demands. Cropping calendars also differ across political regions, which creates a differential ability to respond to climate events (e.g., ENSO), accentuating seasonal differences and changing vulnerability. Predictions of ENSO conditions are skillful in the current generation of seasonal forecast models [44], which translates into information that can be utilized for agriculture management in the Philippines and provides a possibility to mitigate some of the effects of ENSO on rice yields and production. Importantly, extreme ENSO events (such as the 1997/98 El Niño) that lead to large disruptions of the tropical hydroclimate, are projected to occur more frequently by the end of the century in response to greenhouse gas forcing [49]. Thus, the dual calamity of projected changes of both the climate mean state and ENSO-induced hydroclimate variability will likely constitute significant challenges to future rice production in the Philippines.

### Implications for food security in the Philippines

In any given year national production may be adequate, but there might be severe regional shortfalls that impact both food price and security. In the Philippines, regional shortfalls are evident in years when severe natural disasters occurred [42]. Regions of high mean production (S2 Fig) and yield (S3 Fig) naturally dominate the signal seen in national production and yield data (Fig 1 & S1 Fig). However, individual regions and provinces may experience food insecurity that differ from those seen at the national level and can potentially be more severe. The regional relationships between climate variability and production/yield in combination with both the regional long-term mean production/yield and seasonal climate forecasts might help to mitigate future impacts.

Food in the Philippines is relatively mobile, but food prices are more volatile in years with natural disasters [50] and yield shortfalls may disproportionately impact small holders [51]. These negative effects might be mitigated by changing land use patterns, production techniques, or germplasm (breeding material, crop types stored in gene banks, heirloom types, or wild relatives). In the past, land policies in the Philippines have favored expansion of production [52], focusing on increased planting of annual staples [53]. This has led to a steady increase in area under cultivation, including areas that were historically used for other crops. Further, domestic Philippine rice production has been incentivized [53]. As a result, rice yields increased ~1% a year during the second half of the 20^th^ century due to both management and genetics [54], while the area of rice production increased by 50% [16].

Nonetheless, the Philippines are a large importer of rice (~10% of marketed rice per year). This is due geography [55], international policy pressure [56], and colonial history [56], with imports increasing during times of stress (e.g., during the 1997/98 El Niño when rice imports tripled due to fewer harvestable hectares [16]). This has led to calls for self-sufficiency in rice production which, while possible, would be difficult to achieve with current agricultural policy in the Philippines [56] that can leave rice markets susceptible to price increases [20]. It is hypothesized that if there is renewed investment in agriculture, coupled with improved technology and skillful seasonal forecasting, imports could be reduced, helping to increase domestic food security. However, it is unclear if increased investments will provide the necessary buffer to the system to maintain production increases, especially in a changing climate. Additionally, there have been substantial efforts to breed drought resistant rice, with mixed results, due to the trait complexity [57], though new varieties show promise [58].

The north-central area of the Philippines is one of the longest continuously-cultivated areas of rice production in the world. Over time, the objectives of breeding and agronomic endeavors have changed, from local heirloom grown on terraces to mega-varieties grown in an industrial setting across millions of hectares [59]. At the moment, there is increasing interest in heirloom varieties with specific growth environments as a source of both food and export potential [60]. In subsistence settings, rice farming is supplemented by local trade economies that can increase local food security [61]. Moreover, there is a complex agricultural landscape established in the northern Philippines, specifically in Ifugao (rice terraces), where historic intensification has been accompanied by extensification [62]. These examples support the idea that the agro-cultural context can help mitigate the impacts of environmental pressure on food security.

### The role of temperature variability

Our results indicate that temperature variability at present is not a big driver of rice production variability (S4 Fig). Under continued greenhouse gas emissions however, the range of temperature variability in the Philippines is projected to be outside the present-day envelope by the end of the century (Fig 5). Increasing temperatures will have major implications for rice production in the Philippines. Recent work estimated that for every degree Celsius global temperature increase, global mean rice yields will decline by 3.2 ± 3.7% [63]. These reductions were projected without consideration of potential CO_2_ fertilization, adaptation in agronomic practices, or genetic adaptation [63]. While a recent meta-analysis identified an increase in yields under increased CO_2_, this may not be an even increase across crops or regions [64]. Additionally, a comparison between historic and modern cultivars suggests that during modern breeding there has not been a selection for increased response to increased CO_2_ concentrations [65], limiting the potential future CO_2_-fertilization effect.

The temperature sensitivity of crops is dependent on growth stage [66], time of day, and time of year, but generally a temperature increase of one degree can decrease yields by up to 10% once a temperature threshold is reached in rice [67;68;69;70]. Due to this nonlinear threshold behavior, the relative importance of temperature variability to yield variability (S4 Fig) may increase in a warmer climate. The combined effects of high temperatures and moisture deficits could critically alter the seasonality and locality of the impact of ENSO on rice production. Furthermore, by the end of the century, inter-annual climate variability will regularly push climate in the Philippines outside the climatic range of current tropical gene accessions (Fig 5). Most tropical rice accessions currently grow at quarterly temperatures below 28°C. In a 4°C warmer world, median quarterly temperatures will exceed this threshold year-round. In the second quarter in particular, temperatures will already regularly exceed 28°C with just 2°C of global warming. The performance of tropical rice crops in these climatic conditions has not been tested and is thus potentially a large threat to future food security.

### Implications for plant breeding

The ability to increase yields under rising temperatures is a major target for plant breeders [71]. However, modern crop plants have undergone two significant population bottlenecks–the first during domestication and the second during improvement processes–that have resulted in a significant decrease of the crop’s genetic diversity relative to their wild progenitors [72]. For instance, modern Asian rice retains ~80% of the genetic diversity of its wild progenitor [73]. Generally, plant breeding involves crossing 'good by good', a strategy that results in a continuing loss of genetic diversity. Breeding targets focused on yield and quality have often left behind traits from landraces (heirloom lines that have not undergone modern breeding) and crop wild relatives [74]. Among these are many traits associated with tolerance to abiotic stress associated with climate change [75]. There have been increasing efforts to collect data surrounding landrace and wild material in germplasm collections (phenotypes, genotypes, biophysical, environmental) [74], which has led to the creation of a platform to understand the fastest and most practical way to bring in traits from landrace and wild crop material [76]. Breeding is a long-term endeavor, with a long research and development time [77]. This lag time requires a forward-looking approach in order to have plant material ready to be used in the field in time for projected changes in climate. By estimating the current and future temperature envelope of rice production in the Philippines, and comparing this to bioclimatic data of collection locations of rice accessions (Fig 5), we have reduced the number of potential parents that could be used to breed for climate change, thus implementing the first stage of utilizing collections for breeding for climate change.

## Conclusions

There is an increasing need to understand how climate variability will impact rice yields and production, particularly as human population continues to increase and climate changes. Comparing multiple spatial scales allows for a more complete understanding of what types of policy recommendations should be made, as it allows for a direct partitioning into the political units that are most likely to be effective at driving landscape change. This study identified ENSO as driving a significant part of soil moisture variability in the Philippines, which in turn is correlated with rice production and yield variability. Therefore, skillful seasonal predictions can provide useful information for agriculture management to mitigate climate-induced effects on rice production and yield. Future tropical climates is likely to be outside the range of optimal temperatures for rice production. This is true in the Philippines, and will likely require a modification of both genetics and agronomic practices. Detailed case studies like this will complement global yield impact studies and provide important local perspectives.

## Supporting information

S1 Fig National-level rice yields in the Philippines from 1987–2016: Irrigated (blue) and rainfed (red) farming techniques. The linear correlation coefficient R denotes the simultaneous correlation. a) Annual rice yield in the Philippines; b) annual rice yield anomalies (with regard to a 7 yr moving average); c) quarterly rice yield.
(TIF)

S2 Fig Long-term quarterly mean (1987–2016) rice production for both rainfed and irrigated systems. Note that grid point values indicate the mean production value of the whole associated province.
(TIF)

S3 Fig Long-term quarterly mean (1987–2016) rice yield for both rainfed and irrigated systems. (TIF)

S4 Fig Correlation coefficient R between and quarterly rice yield and surface temperature anomalies in the previous quarter. The annual cycle is removed and yield anomalies are with regard to a 7 yr moving average. The temperature data are area averaged for each political region corresponding to the rice yield data.
(TIF)

S1 Table The table shows if rice is planted or harvested in the administrative regions of the Philippines according the PhilRice planting calendar. (DOCX)

## References

[1] Khoury CK., Bjorkman AD., Dempewolf H, Ramirez-Villegas J, Guarino L, Jarvis A.et al Increasing homogeneity in global food supplies and the implications for food security. PNAS, 2014; 111, 4001–4006. 10.1073/pnas.1313490111 24591623 PMC3964121

[2] Ray DK, Mueller ND, West PC, Foley JA. Yield trends are insufficient to double global crop production by 2050. PloS one, 2013; 8, e66428 10.1371/journal.pone.0066428 23840465 PMC3686737

[3] Muthayya S, Sugimoto JD, Montgomery S, Maberly GF. An overview of global rice production, supply, trade, and consumption. Annals of the New York Academy of Sciences, 2014; 1324, 7–14. 10.1111/nyas.12540 25224455

[4] Bersales, LG. Republic of Philippines. Philippine Statistics Authority. Selected Statistics on Agriculture 2016. ISSN-2012-0362. Quezon City, Philippines. Philippine Statistics Authority 2016

[5] Haefele SM, Nelson A, Hijmans RJ. Soil quality and constraints in global rice production. Geoderma, 2014; 235, 250–259.

[6] Cardwell VB. Fifty years of Minnesota corn production: sources of yield increase. Agron. J., 1982; 74, 984–990.

[7] Fischer RA. Definitions and determination of crop yield, yield gaps, and of rates of change. Field Crops Research, 2015; 182, 9–18.

[8] Specht JE, Hume DJ, Kumudini SV. Soybean yield potential—a genetic and physiological perspective. Crop Science, 1999; 39, 1560–1570.

[9] Tollenaar M, Lee EA. Yield potential, yield stability and stress tolerance in maize. Field Crops Research, 2002; 75, 161–169.

[10] Bell MA, Fischer RA, Byerlee D, Sayre K. Genetic and agronomic contributions to yield gains: A case study for wheat. Field Crops Research, 1995; 44, 55–65.

[11] Lesk C, Rowhani P, Ramankutty N. Influence of extreme weather disasters on global crop production. Nature, 2016; 529, 84–87. 10.1038/nature16467 26738594

[12] Battisti DS, Naylor RL. Historical warnings of future food insecurity with unprecedented seasonal heat. *Science*, 2009; 323, 240–244. 10.1126/science.1164363 19131626

[13] Urban DW, Sheffield J, Lobell DB. The Impacts of Future Climate and Carbon Dioxide Changes on the Average and Variability of US Maize Yields under Two Emission Scenarios. Environmental Research Letters, 2015; 10, 045003.

[14] Iizumi T, Ramankutty N. Changes in yield variability of major crops for 1981–2010 explained by climate change. Environmental Research Letters, 2016; 11, p.034003.

[15] Fedoroff NV, Battisti DS, Beachy RN, Cooper PJ, Fischhoff DA et al Radically rethinking agriculture for the 21st century. Science, 2010; 27, 833–834. 10.1126/science.1186834 PMC3137512 20150494

[16] FAO. 2014. FAOSTAT, Production. Available at http://faostat3.fao.org/home/E. Accessed July 2016.

[17] Foley JA, Ramankutty N, Brauman KA, Cassidy ES, Gerber JS, Johnston M, et al 2011. Solutions for a cultivated planet. Nature, 2011; 478, 337–342. 10.1038/nature10452 21993620

[18] Tilman D, Balzer C, Hill J, Befort BL. Global food demand and the sustainable intensification of agriculture. PNAS, 2011; 108, 20260–20264. 10.1073/pnas.1116437108 22106295 PMC3250154

[19] Koide N, Robertson AW, Ines AV, Qian JH, DeWitt DG, Lucero A. Prediction of rice production in the Philippines using seasonal climate forecasts. Journal of Applied Meteorology and Climatology, 2013; 52,552–569.

[20] Koirala KH, Mishra A, Mohanty S. Impact of land ownership on productivity and efficiency of rice farmers: The case of the Philippines. Land Use Policy, 2016; 50, 371–378.

[21] Lampayan RM., Palis FG, Soriano JB, Bouman BAM. 2016. Farmers’ participatory research and adoption of aerobic rice in the Philippines. Regional: Development and Dissemination of Climate-Resilient Rice Varieties for Water-Short Areas of South Asia and Southeast Asia, 2016; 315.

[22] Rasmusson EG, Carpenter TH. Variations in Tropical Sea Surface Temperature and Surface Wind Fields Associated with the Southern Oscillation/El Niño. Monthly Weather Review, 1982; 110, 354–384.

[23] McPhaden MJ, Zebiak SE, Glantz MH. ENSO as an integrating concept in Earth science. Science, 2006; 314, 1740–1745. 10.1126/science.1132588 17170296

[24] Stuecker MF, Timmermann A, Jin F-F, McGregor S, Ren H-L. A combination mode of the annual cycle and the El Niño/Southern Oscillation. Nature Geoscience, 2013; 6, 540–544.

[25] Roberts MG, Dawe D, Falcon WP, Naylor RL. El Niño-Southern Oscillation impacts on rice production in Luzon, the Philippines. Journal of Applied Meteorology and Climatology, 2009; 48, 1718–1724.

[26] Iizumi T, Luo JJ, Challinor AJ, Sakurai G, Yokozawa M, Sakuma H, Sakuma H, et al Impacts of El Niño Southern Oscillation on the global yields of major crops. Nature Communications, 2014; 5. 10.1038/ncomms4712 24827075

[27] Naylor RL, Falcon WP, Rochberg D, Wada N. Using El Nino/Southern Oscillation climate data to predict rice production in Indonesia. Climatic Change, 2001; 50, 255–265.

[28] Naylor RL, Battisti DS, Vimont DJ, Falcon WP, Burke MB. Assessing risks of climate variability and climate change for Indonesian rice agriculture. PNAS, 2007; 104, 7752–7757. 10.1073/pnas.0701825104 17483453 PMC1876519

[29] Osborne T, Rose G, Wheeler T. Variation in the global-scale impacts of climate change on crop productivity due to climate model uncertainty and adaptation. Agricultural and Forest Meteorology, 2013; 170, 183–194.

[30] Lipper L, Thornton P, Campbell BM, Baedeker T, Braimoh A, Bwalya M, Caron P. et al Climate-smart agriculture for food security. Nature Climate Change, 2014; 4, 1068–1072.

[31] Hijmans RJ, Cameron SE, Parra JL, Jones PG, Jarvis A. Very high resolution interpolated climate surfaces for global land areas. International Journal of Climatology, 2005; 25: 1965–1978.

[32] Kucharik CJ, Ramankutty N. Trends and variability in US corn yields over the twentieth century. Earth Interactions, 2005; 9, 1–29.

[33] Bernardo R. Essentials of plant breeding. Stemma Press Woodbury, MN 2014

[34] Rayner NA, Parker DE, Horton EB, Folland CK, Alexander LV, Rowell DP. Global analyses of sea surface temperature, sea ice, and night marine air temperature since the late nineteenth century, J. Geophys. Res., 2003; 108, 4407.

[35] Fan Y, van den Dool H. Climate Prediction Center global monthly soil moisture data set at 0.5° resolution for 1948 to present, J. Geophys. Res., 2004; 109, D10102

[36] Dee DP, Uppala SM, Simmons AJ, Berrisford P, Poli P, Kobayashi S et al The ERA-Interim reanalysis: configuration and performance of the data assimilation system. Q.J.R. Meteorol. Soc., 2011; 137, 553–597.

[37] University of East Anglia Climatic Research Unit, Harris IC, Jones PD. CRU TS3.23: Climatic Research Unit (CRU) Time-Series (TS) Version 3.23 of High Resolution Gridded Data of Month-by-month Variation in Climate (Jan. 1901- Dec. 2014) [Internet]. Centre for Environmental Data Analysis; 2015. 10.5285/4c7fdfa6-f176-4c58-acee-683d5e9d2ed5

[38] Taylor KE, Stouffer RJ, Meehl GA. An Overview of CMIP5 and the experiment design. Bull. Amer. Meteor. Soc., 2012; 93, 485–498.

[39] Tebaldi C, Arblaster JM. Pattern scaling: Its strengths and limitations, and an update on the latest model simulations. *Clim Change*. 2014 122(3):459–471.

[40] Tigchelaar M, Battisti DS, Naylor RL, Ray DK. Future warming increases probability of globally synchronized maize production shocks. 2018. PNAS 2018; 10.1073/pnas.1718031115 29891651 PMC6042138

[41] Huigen MG, Jens IC. Socio-economic impact of super typhoon Harurot in San Mariano, Isabela, the Philippines. World Development, 2006; 34, 2116–2136.

[42] Israel, DC, Briones, RM. Impacts of natural disasters on agriculture, food security, and natural resources and environment in the Philippines (No. 2012–36). PIDS discussion paper series. 2012

[43] Bagtasa G. Contribution of Tropical Cyclones to Rainfall in the Philippines. *J*. *Climate*. 2017; 10.1175/JCLI-D-16-0150.1

[44] Barnston AG, Tippett MK, van den Dool HM, Unger DA. Toward an Improved Multimodel ENSO Prediction. J. Appl. Meteor. Climatol., 2015; 54, 1579–1595, 10.1175/JAMC-D-14-0188.1

[45] Zwart SJ, Bastiaanssen WG. Review of measured crop water productivity values for irrigated wheat, rice, cotton and maize. Agricultural water management, 2004; 69, 115–133.

[46] Atwell BJ, Wang H, Scafaro AP. Could abiotic stress tolerance in wild relatives of rice be used to improve Oryza sativa? Plant Science, 2014; 215, 48–58. 10.1016/j.plantsci.2013.10.007 24388514

[47] Cassman KG. Ecological intensification of cereal production systems: yield potential, soil quality, and precision agriculture. PNAS, 1999; 96, 5952–5959. 10339523 10.1073/pnas.96.11.5952 PMC34211

[48] Lobell DB, Burke MB. Why are agricultural impacts of climate change so uncertain? The importance of temperature relative to precipitation. Environmental Research Letters, 2008; 3, 034007.

[49] Cai W, Santoso A, Wang G, Yeh SW, An SI, Cobb KM, et al ENSO and greenhouse warming. Nature Climate Change. 2015; 5(9):849.

[50] Dawe D, Maltsoglou I. Marketing margins and the welfare analysis of food price shocks. Food Policy, 2014; 46, 50–55.

[51] Samberg LH, Gerber JS, Ramankutty N, Herrero M, West PC. Subnational distribution of average farm size and smallholder contributions to global food production. Environmental Research Letters, 2016; 11, 124010.

[52] Lapar MLA, Pandey S. Adoption of soil conservation: the case of the Philippine uplands. Agricultural economics, 1999; 21: 241–256.

[53] Coxhead I, Shively G, Shuai X. Development policies, resource constraints, and agricultural expansion on the Philippine land frontier. Environment and Development Economics, 2002; 7, 341–363.

[54] Peng S, Laza RC, Visperas RM, Sanico AL, Cassman KG, Khush GS. Grain yield of rice cultivars and lines developed in the Philippines since 1966. Crop Science, 2000; 40:2, 307–314.

[55] Dawe DC, Moya P, Casiwan CB. (Eds.). Why does the Philippines import rice?: meeting the challenge of trade liberalization Int. Rice Res. Institute. Los Banos, Philippines 2006

[56] Davidson JS. Why the Philippines chooses to import rice. Critical Asian Studies, 2016; 48, 100–122.

[57] Serraj R, McNally KL, Slamet-Loedin I, Kohli A, Haefele SM, Atlin G, et al 2011 Drought resistance improvement in rice: an integrated genetic and resource management strategy. Plant Production Science, 14(1), 1–14.

[58] Ohno H, Banayo NP, Bueno C, Kashiwagi JI, Nakashima T, Iwama K, et al 2018 On-farm assessment of a new early-maturing drought-tolerant rice cultivar for dry direct seeding in rainfed lowlands. Field Crops Research, 219, 222–228.

[59] Glover D, Stone GD. 2018 Heirloom rice in Ifugao: an ‘anti-commodity’in the process of commodification. The Journal of Peasant Studies, 45(4), 776–804.

[60] Stone GD, Glover D. 2017 Disembedding grain: Golden Rice, the Green Revolution, and heirloom seeds in the Philippines. Agriculture and Human Values, 34(1), 87–102.

[61] Longacre WA, Hermes TR. 2015 Rice farming and pottery production among the Kalinga: New ethnoarchaeological data from the Philippines. Journal of Anthropological Archaeology, 38, 35–45.

[62] Acabado S. 2012 The Ifugao agricultural landscapes: Agro-cultural complexes and the intensification debate. Journal of Southeast Asian Studies, 43(3), 500–522.

[63] Zhao C, Liu B, Piao S, Wang X, Lobell DB, Huang Y, et al 2017 Temperature increase reduces global yields of major crops in four independent estimates. PNAS, 2017; 114(35), 9326–9331. 10.1073/pnas.1701762114 28811375 PMC5584412

[64] Ainsworth EA. Rice production in a changing climate: a meta-analysis of responses to elevated carbon dioxide and elevated ozone concentration. Global Change Biology, 2008; 14, 1642–1650.

[65] Franzaring J, Holz I, Fangmeier A. Responses of old and modern cereals to CO2-fertilisation. Crop and Pasture Science; 2014; 64, 943–956.

[66] Lansigan FP, De los Santos WL, Coladilla JO. Agronomic impacts of climate variability on rice production in the Philippines. Agriculture, ecosystems & environment, 2000; 82, 129–137.

[67] Peng S, Huang J, Sheehy JE, Laza RC, Visperas RM, Zhong X, et al Rice yields decline with higher night temperature from global warming. PNAS, 2004; 101, 9971–9975. 10.1073/pnas.0403720101 15226500 PMC454199

[68] Luo Q. Temperature thresholds and crop production: a review. Climatic Change, 2011; 109, 583–598.

[69] Krishnan P, Swain DK, Bhaskar BC, Nayak SK, Dash RN. Impact of elevated CO2 and temperature on rice yield and methods of adaptation as evaluated by crop simulation studies. Agriculture, Ecosystems & Environment, 2007; 122, 233–242.

[70] Sheehy JE, Mitchell PL, Ferrer AB. Decline in rice grain yields with temperature: models and correlations can give different estimates. Field Crops Research, 2006; 98, 151–156.

[71] Dempewolf H, Eastwood RJ, Guarino L, Khoury CK, Müller JV, Toll J. Adapting agriculture to climate change: a global initiative to collect, conserve, and use crop wild relatives. Agroecology and Sustainable Food Systems, 2014; 38, 369–377.

[72] Kantar MB, Nashoba AR, Anderson JE, Blackman BK, Rieseberg LH. The Genetics and Genomics of Plant Domestication. BioScience, BioScience, 2017; 67, 971–982.

[73] Huang X, Kurata N, Wang ZX, Wang A, Zhao Q, Zhao Y, et al A map of rice genome variation reveals the origin of cultivated rice. Nature, 2012; 490, 497–501. 10.1038/nature11532 23034647 PMC7518720

[74] Dempewolf H, Baute G, Anderson J, Kilian B, Smith C, Guarino L. Past and Future Use of Wild Relatives in Crop Breeding. Crop Sci, 2017; 57, 1–13.

[75] Anderson JE, Kono TJ, Stupar RM, Kantar MB, Morrell PL. Environmental association analyses identify candidates for abiotic stress tolerance in Glycine soja, the wild progenitor of cultivated soybeans. G3, 2016; 6, 835–843. 10.1534/g3.116.026914 26818076 PMC4825654

[76] Prohens J, Gramazio P, Plazas M, Dempewolf H, Kilian, et al Introgressiomics: a new approach for using crop wild relatives in breeding for adaptation to climate change. Euphytica, 2017; 213, 158.

[77] Pardey P. G., Chan-Kang C., Dehmer S.P., Beddow J. M. 2016 Agricultural R&D is on the Move. Nature, 537, 301–303 10.1038/537301a 27629624

[78] Plant genetic resources accession level data provided by: USDA ARS NPGS, CGIAR, EURISCO, and other data providers to Genesys. All intellectual property rights (including copyright) in the Data are owned and retained by the said institution(s). Data accessed through GENESYS Global Portal on Plant Genetic Resources, http://www.genesys-pgr.org, 2017-03-15.

