# Supplementary figures and images for: Climate variability impacts on rice production in the Philippines
Source: PLoS One. 2018 Aug 9;13(8):e0201426. doi: 10.1371/journal.pone.0201426 (PMC6084865; doi:10.1371/journal.pone.0201426)

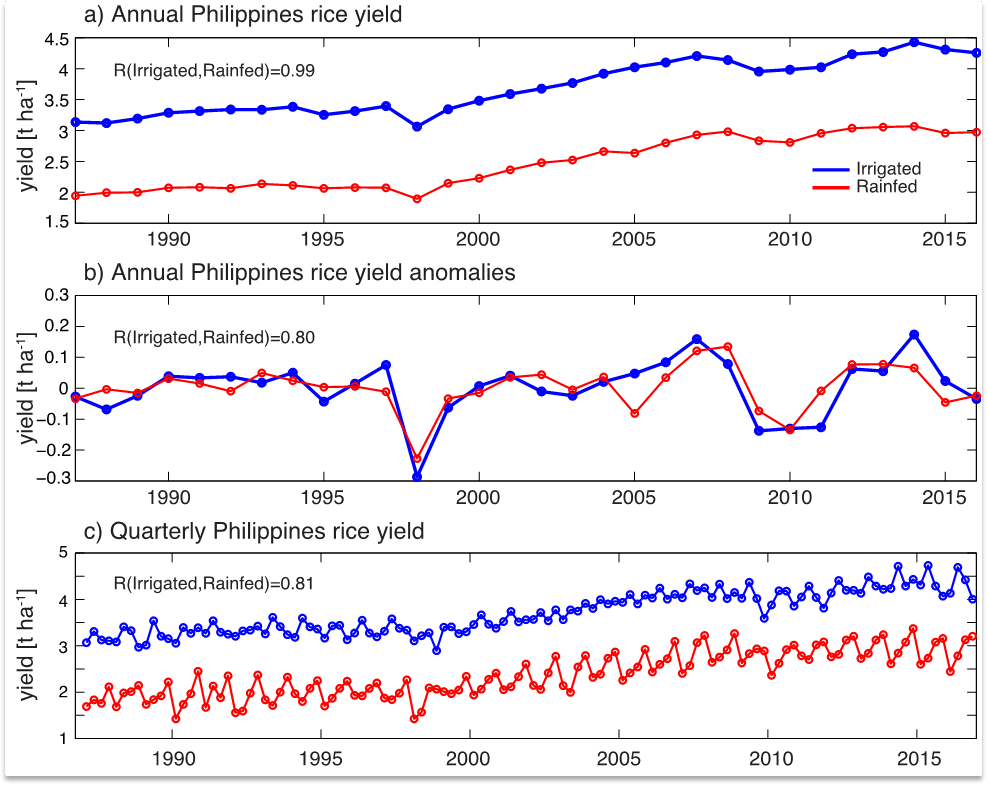

Supplement: S1 Fig — The linear correlation coefficient R denotes the simultaneous correlation. a) Annual rice yield in the Philippines; b) annual rice yield anomalies (with regard to a 7 yr moving average); c) quarterly rice yield. (TIF) [file pone.0201426.s001.tif]

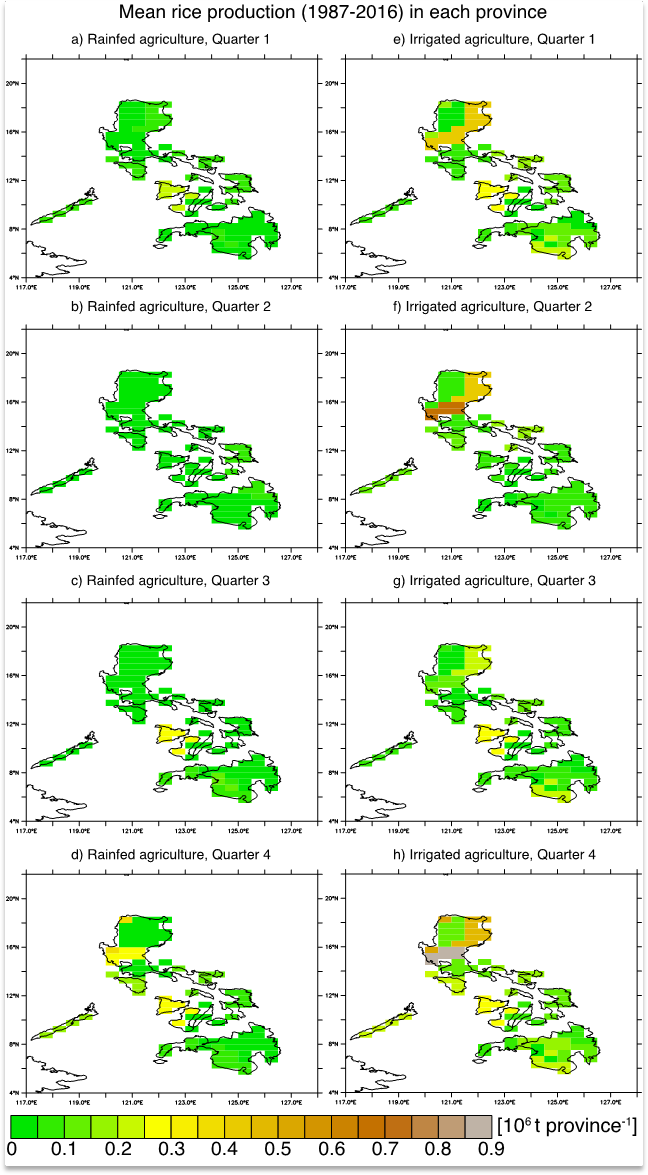

Supplement: S2 Fig — Note that grid point values indicate the mean production value of the whole associated province. (TIF) [file pone.0201426.s002.tif]

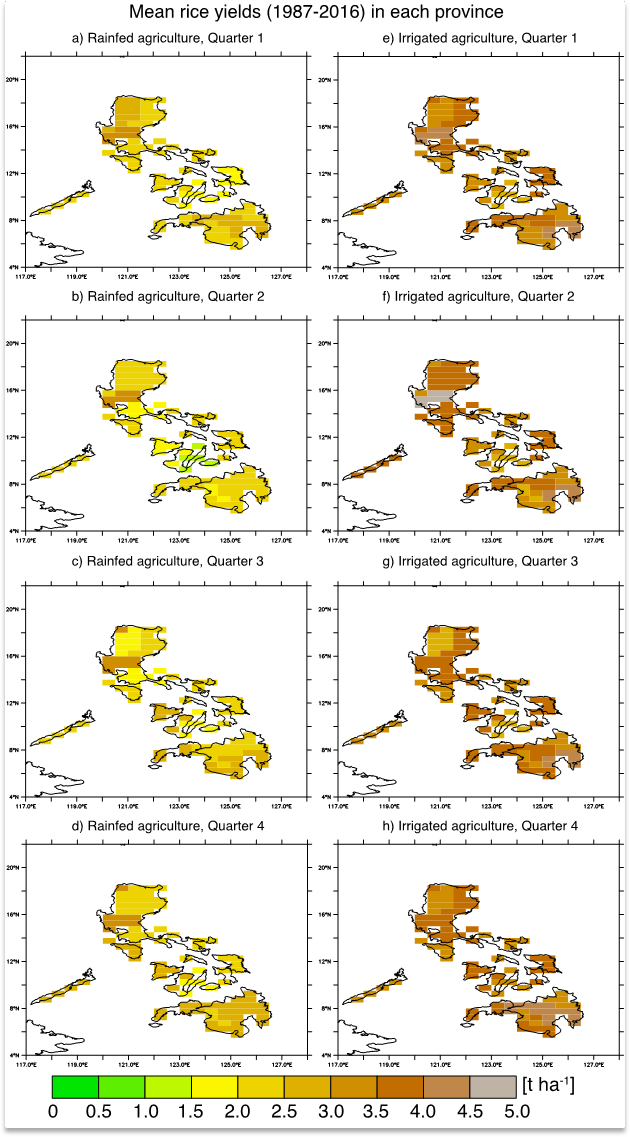

Supplement: S3 Fig — (TIF) [file pone.0201426.s003.tif]

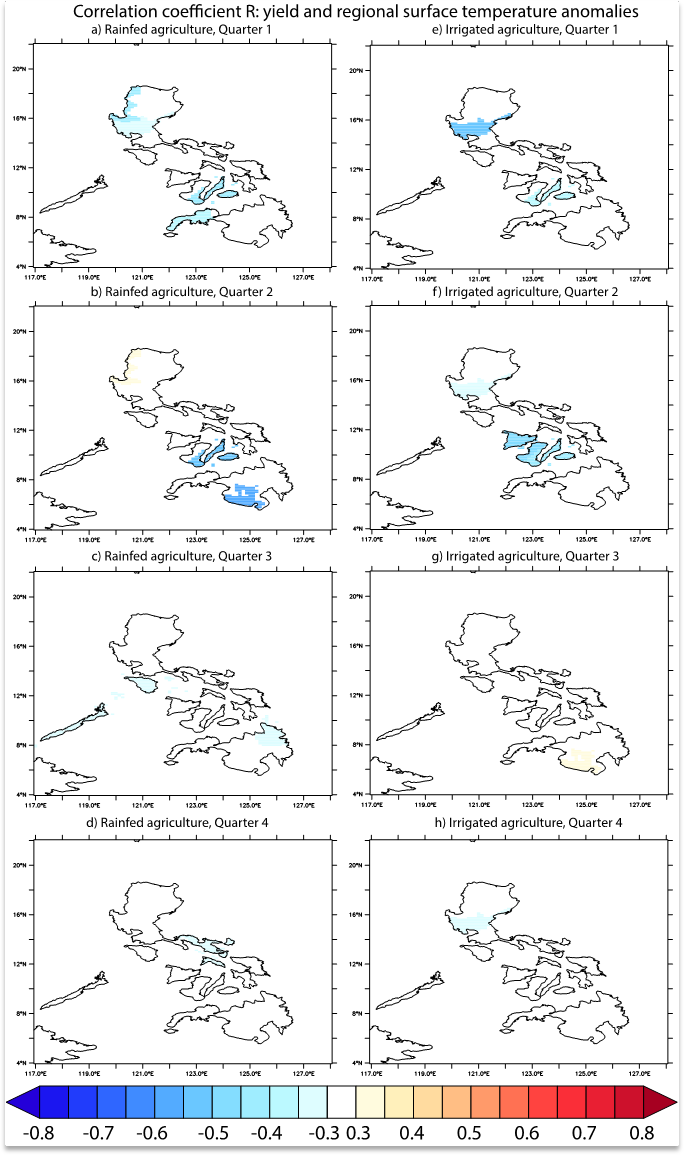

Supplement: S4 Fig — The annual cycle is removed and yield anomalies are with regard to a 7 yr moving average. The temperature data are area averaged for each political region corresponding to the rice yield data. (TIF) [file pone.0201426.s004.tif]
