# Supplementary material for: Climate variability impacts on rice production in the Philippines
Source: PLoS One. 2018 Aug 9;13(8):e0201426. doi: 10.1371/journal.pone.0201426 (PMC6084865; doi:10.1371/journal.pone.0201426)
Supplement: S1 Table — (DOCX) [file pone.0201426.s005.docx]

**Table S1.** The table shows if rice is planted or harvested in the administrative regions of the Philippines according the PhilRice planting calendar.

| **Region** | **Q1_Plant** | **Q1_Harvest** | **Q2_Plant** | **Q2_Harvest** | **Q3_Plant** | **Q3_Harvest** | **Q4_Plant** | **Q4_Harvest** |
| --- | --- | --- | --- | --- | --- | --- | --- | --- |
| NCR | NA | NA | NA | NA | NA | NA | NA | NA |
| CAR | Yes | Yes | Yes | Yes | Yes | Yes | Yes | Yes |
| ILOCOS REGION | Yes | Yes | Yes | Yes | Yes | Yes | Yes | Yes |
| CAGAYAN VALLEY | No | Yes | Yes | Yes | Yes | Yes | Yes | No |
| CENTRAL LUZON | Yes | Yes | Yes | Yes | Yes | Yes | Yes | Yes |
| CALABARZON | Yes | Yes | Yes | Yes | Yes | Yes | Yes | Yes |
| MIMAROPA | Yes | Yes | Yes | Yes | Yes | Yes | Yes | Yes |
| BICOL REGION | Yes | Yes | Yes | Yes | Yes | Yes | Yes | Yes |
| WESTERN VISAYAS | NA | NA | NA | NA | NA | NA | NA | NA |
| CENTRAL VISAYAS | NA | NA | NA | NA | NA | NA | NA | NA |
| EASTERN VISAYAS | Yes | Yes | Yes | Yes | Yes | Yes | Yes | Yes |
| ZAMBOANGA PENINSULA | NA | NA | NA | NA | NA | NA | NA | NA |
| NORTHERN MINDANAO | Yes | Yes | Yes | Yes | Yes | Yes | Yes | Yes |
| DAVAO REGION | No | Yes | Yes | No | No | Yes | Yes | No |
| SOCCSKSARGEN | NA | NA | NA | NA | NA | NA | NA | NA |
| CARAGA | Yes | Yes | Yes | Yes | Yes | Yes | Yes | Yes |
| ARMM | NA | NA | NA | NA | NA | NA | NA | NA |
